# Relationship between potentially toxic elements and macrophyte communities in the Sava river

**DOI:** 10.1016/j.heliyon.2024.e34994

**Published:** 2024-07-20

**Authors:** Snežana Jarić, Branko Karadžić, Momir Paunović, Radmila Milačič, Janez Ščančar, Olga Kostić, Tea Zuliani, Janja Vidmar, Zorana Miletić, Stefan Anđus, Miroslava Mitrović, Pavle Pavlović

**Affiliations:** a Department of Ecology, Institute for Biological Research ‘Siniša Stanković’ University of Belgrade, Bul. Despota Stefana 142, 11060 Belgrade, Serbia; b Department of Hydroecology and Water Protection, Institute for Biological Research ‘Siniša Stanković’ University of Belgrade, Bul. Despota Stefana 142, 11060 Belgrade, Serbia; c Department of Environmental Sciences, Jožef Stefan Institute, Jamova cesta 39, 1000 Ljubljana, Slovenia

**Keywords:** Aquatic vegetation, Biodiversity, Heavy metals, Potentially toxic elements, Sava river, Macrophyte communities

## Abstract

Freshwater ecosystems are at significant risk of contamination by potentially toxic elements (PTEs) due to their high inherent toxicity, their persistence in the environment and their tendency to bioaccumulate in sediments and living organisms. We investigated aquatic macrophyte communities and the concentrations of As, Cu, Cd, Cr, Pb, Zn, Ni and Fe in water and sediment samples to identify a pollution pattern along the Sava River and to investigate the potential impact of these PTEs on the diversity and structure of macrophyte communities. The study, which covered 945 km of the Sava River, showed a downstream increase in sediment concentrations of the analyzed elements. Both species richness and alpha diversity of macrophyte communities also generally increase downstream. Ordinary and partial Mantel tests indicate that macrophyte communities are significantly correlated with sediment chemistry, but only weakly correlated with water chemistry. In the lowland regions (downstream), beta diversity decreases successively, which can be attributed to an increasing similarity of environmental conditions at downstream sites. Species richness is relatively low at sites with low concentrations of Cr, Cd, Fe, and Cu in the sediment. However, species richness increases to a certain extent with increasing element concentrations; as element concentrations increase further, species richness decreases, probably as a result of increased toxicity. Some species that are generally more tolerant to high concentrations of PTEs are: *Ceratophyllum demersum, Iris pseudacorus, Najas marina, Butomus umbellatus, Vallisneria spiralis, Potamogeton gramineus* and *Bolboschoenus maritimus maritimus*. *Potamogeton perfoliatus* and the moss species *Cinclidotus fontinaloides* and *Fontinalis antipyretica* have narrow ecological amplitudes in relation to the concentrations of PTEs in the sediment.

## Highlights

• The effect of trace elements on aquatic vegetation along the Sava River was studied.
• Concentrations of analyzed elements increase in the downstream direction.
• Species richness and alpha diversity also increase in the downstream direction.
• There is a significant correlation between sediment pollution and species richness of macrophytes.
• Elevated Cr, Cd, Fe, and Cu concentrations can affect species richness.

## 1 Introduction

Human activity exposes freshwater ecosystems to wide spectra of natural and anthropogenic pollution that affect their function through changes in species diversity, especially when the ecosystem processes (e.g. primary production) are dependent on a small number of species [[1], [2], [3]]. Abiotic and biotic factors do not usually act independently, but rather interact, creating synergetic effects on the biodiversity and functioning of aquatic ecosystems via changes in the physiology of individual species and population traits, causing disturbances in community balance [3].

Anthropogenic pressure on water resources is increasing worldwide and is the subject of numerous studies [2,[4], [5], [6], [7]]. Knowledge of the complex response of hydrological (e.g. hydropeaking, water scarcity, flooding), geochemical (e.g. chemical pollution and erosion), and ecological (e.g. invasive species emergence, biodiversity decline) in large river basins (10.000 km2 or more) is still incomplete. Additional research is needed to enable more effective management of water resources at the catchment scale [[7], [8], [9], [10]]. As river pollution knows no national borders, it is essential to continuously monitor pollutants in water, sediment, soil, and aquatic biota to gain a comprehensive overview of the pollution levels in the river basin. The sources of pollution are manifold and it is difficult to precisely identify the multiple discharge points, e.g. the discharge of urban effluent, industrial effluent, excavated mining waste, fertilizers, and pesticides [[10], [11], [12]]. In addition, pollution can be affected by background geochemical loads, which can be considerable in regions where the parent rock layers contain hazardous and basin-specific pollutants, such as potentially toxic elements (PTEs) [13].

Macrophytes are an important component of aquatic ecosystems. Potential changes in their number, as well as in the composition of the communities themselves, can offer an answer to the question how and why an ecosystem changes. The survival of aquatic plants is affected by numerous environmental factors. These include rapid water flow, fluctuating water levels, the availability of light, hypoxic conditions and insufficient concentrations of essential minerals for metabolism [14,15]. However, macrophytes have the ability to adapt to the cumulative effects of anthropogenic pollution over a long period of time (measured in years), which is helpful to the study of the ecological status of a river [16,17]. The metabolic processes of macrophytes play a decisive role in the regulation of biogeochemical cycles in aquatic ecosystems. Namely, macrophytes are highly tolerant to multiple stressors (hydrology, nutrient loading, morphological alterations) because of broad ecological plasticity conditioned to taxonomic diversity and the diversity of life forms [17,18]. For this reason, macrophytes are gaining importance as an important tool for water quality monitoring. Additionally, aquatic plants, with their selective absorption of certain ions and sedentary nature, are suitable biological monitors in water quality studies focused on PTEs and other pollutants in the water and submerged soil [19,20].

The Sava River is one of the most important waterways in southeastern Europe, and its basin is the Balkan Peninsula's main connection with the western parts of Europe. Anthropogenic pressures on the aquatic ecosystems of the Sava are numerous. The anthropogenic pressure on water and wetland ecosystems along the Sava River is primarily due to intensive urbanization and the development of various economic sectors, including energy, mining, industry, agriculture and transport. The upper course of the river is exposed to hydromorphological pressures, the middle to agricultural activities and eutrophication, and the lower course mostly to industrial and urban pollution [[21], [22], [23]]. The dense network of industrial cities along the Sava River requires a continuous supply of extensive energy resources. Power generation along the river is made possible by the Krško nuclear power plant, numerous hydroelectric power plants on the Sava and its tributaries, and thermoelectric power plants in Obrenovac. These power generation plants rely on various natural resources (fossil fuels, water, radioactive material) and consequently cause unavoidable environmental impacts. The environmental impact varies depending on the specific technologies used in the energy sector.

The river Sava crosses numerous cities with established industrial plants. Municipal and industrial wastewater discharged along the Sava River and its tributaries is often not properly treated and poses a significant threat to water quality. The main pollutants include organic waste, persistent organic pollutants (POPs), PTEs, fertilizers and radioactive elements, all of which have harmful effects on human health, biodiversity and the environment [[24], [25], [26]]. In recent years, the interest of researchers in this problem has grown [[26], [27], [28], [29], [30], [31], [32]]. Consequently, chemical pollution from diffuse sources has been identified by current European legislation as one of the main factors affecting the quality of rivers. A prerequisite for effective river management is a full appreciation of how the surrounding landscape influences the structure and functioning of a river ecosystem. This aspect is included in the EU Water Framework Directive [33] where macrophytes are described as an important biological component which is required for the determination of the ecological status of flowing water. Deterioration of the physical stream environment and eutrophication brings about changes in macrophyte distribution, causing a decline in macrophyte species richness and increased abundance of more resistant species [[34], [35], [36], [37]]. This is very important since macrophyte assemblages are considered to be an important component of fluvial ecosystems. Therefore, the main aims of this study were to: (i) screen the total concentrations of eight chemical elements (As, Cu, Cd, Cr, Pb, Zn, Ni and Fe) in water and sediment samples, and to (ii) detect, determine and quantify (abundance) macrophytes.

## 2 Material and methods

### 2.1 Study area

**Fig. 1:**
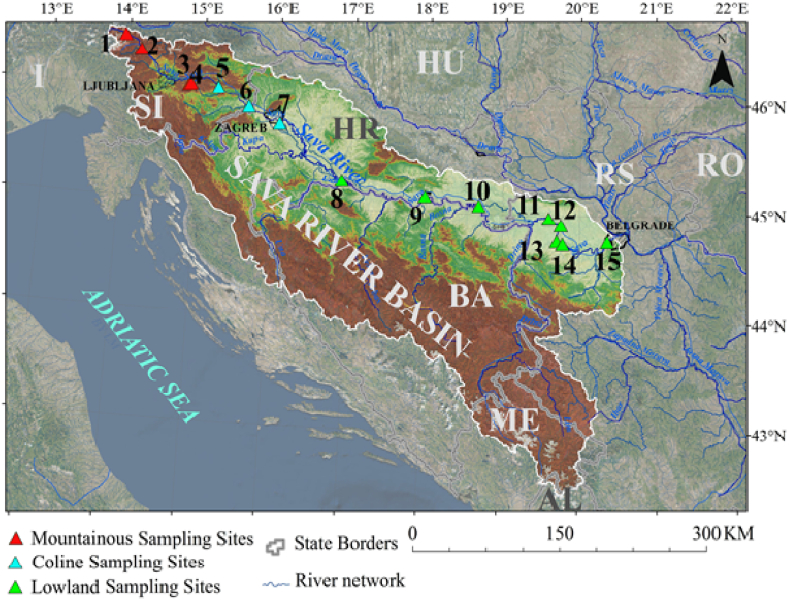
Map of the Sava River basin with sampling sites (labelled 1–15, see Table 1).

The Sava River is formed by the Sava Dolinka (45-km long) and Sava Bohinjka (31-km long) rivers, and represents the largest drainage basin of southeastern Europe: it is the biggest tributary to the Danube River. It is 945 km long, and the total catchment area is almost 97,713.20 km^2^. The location of the Sava River basin is between the longitudes 13.67°E and 20.58°E and between latitudes 42.43°N and 46.52°N: its surface is shared by six countries: Slovenia, Croatia, Bosnia and Herzegovina, Serbia, Montenegro and Albania (rising in Slovenia and flowing through Croatia and Serbia, where it discharges into the Danube). The hydrographic network of the basin is well developed (Fig. 1., [38]). The population of the area of the river basin is almost 8,176,000, which is 46 % of the total number of inhabitants of the abovementioned countries (without Albania and Montenegro) [39]. The Sava River basin is characterized by overall very heterogeneous environmental conditions. An alpine climate dominates the upper part of the basin in Slovenia; in the areas of the basin tributaries on the right (Croatia, Bosnia and Herzegovina and Montenegro) the climate is moderate continental; a moderate-continental Central European-type climate primarily characterizes the basin area of the left tributaries that belong to the Pannonian basin. The average annual temperature of the whole river basin is almost 9.5 °C, the average annual precipitation is almost 1100 mm, and average annual evapotranspiration is almost 530 mm [38]. The altitude of the River Sava ranges from 71 m a.s.l. at its confluence with the River Danube (Belgrade, Serbia), to 2864 m a.s.l. at the top of Mt. Triglav in the Slovenian Alps. The average altitude of the catchment area is around 545 m a.s.l., and according to FAO (Food and Agriculture Organization) classification, the predominant slope in the basin is moderately steep (the mean is 15.8 %).

The study was carried out along the River Sava within the framework of the GLOBAQUA project (2014–2019) [6]. Fifteen different sampling stretches were selected: Mojstrana (MOJ), Radovljica (RAD), Litija − upstream (LIT1), Litija − downstream (LIT2), Vrhovo (VRH) and Čatež (CAT) in Slovenia, Zagreb (ZAG), Jasenovac (JAS), Slavonski Brod (SLB) and Županja (ZUP) in Croatia, Sremska Mitrovica − upstream (SRM1), Sremska Mitrovica − downstream (SRM2), Šabac − upstream (SAB1), Šabac – downstream (SAB2) and Belgrade (BEO) in Serbia (Fig. 1, Table 1). Characteristics of the sampling sites are presented in Table 1. The samples were divided into three groups: mountainous sites that were sampled in upper course of the Sava River, sub-mountainous or colline sites along the middle course, and lowland sites along the peri-Pannonian plain.

**Table 1:** Sampling sites along the Sava River.

Site No.	Site name (abbreviations)	Region	Altitude	Global coordinates		Distance from source (km)
				North	East	
1.	Mojstrana (MOJ)	mountainous	661	46.4599	13.9400	930
2.	Radovljica (RAD)	mountainous	406	46.2929	14.2605	900
3.	Litija - upstream (LIT1)	mountainous	230	46.0556	14.8231	819
4.	Litija - downstream (LIT2)	mountainous	225	46.0660	14.8504	810
5.	Vrhovo (VRH)	colline	194	46.0453	15.2153	750
6.	Čatez (CAT)	colline	134	45.8549	15.6944	729
7.	Zagreb (ZAG)	colline	110	45.7569	16.0457	640
8.	Jasenovac (JAS)	lowland	87	45.2636	16.8942	500
9.	Slavonski Brod (SLB)	lowland	81	45.1262	18.0847	348
10.	Županja (ZUP)	lowland	76	45.0150	18.7398	249
11.	Sremska Mitrovica -upstream (SRM1)	lowland	73	44.9730	19.5961	139
12.	Sremska Mitrovica - downstream (SRM2)	lowland	72	44.9135	19.7524	118
13.	Šabac - upstream (SAB1)	lowland	71	44.7699	19.6994	106
14.	Šabac - downstream (SAB2)	lowland	71	44.7449	19.7789	99
15.	Belgrade (BEO)	lowland	69	44.7685	20.3555	14

### 2.2 Sampling

*Macrophyte sampling –* The sampling of macrophytes was carried out according to the Protocol on Sampling of Macrophytes in accordance with the GLOBAQUA project [6]. In the shallow parts of the river basin, sampling was conducted upstream on a zig-zag walk through the river, and where such a walk was not safe, a boat was used to sail upstream in a zig-zag manner or along the left and right banks, observing from the shore (length 1 river kilometer in each locality). Macrophyte research on the sampling sites is based on the detection, determination, quantification (abundance) and sampling required for the analysis of the concentration of PTEs in selected macrophyte species. The abundance of each plant species was recorded according to the 5-point scale devised by Kohler [40]: 1 = very rare, 2 = rare, 3 = common, 4 = frequent, 5 = abundant, predominant. The degree of similarity of the macrophyte flora recorded in the River Sava with the macrophytic communities in other rivers was calculated using the Sørensen similarity index [41]:

(1) Cs = 2xC/(A + B)x100

A – total number of macrophytic species of one river
B – total number of macrophytic species of another river
C− number of shared macrophytes
C_S_ – Sørensen similarity index

The correct taxonomy and nomenclature of the vascular plants was checked referring to World Flora Online (WFO) (www.worldfloraonline.org).

*Water and sediment sampling* − Water and sediment samples were samples at each site for detailed chemical analyses. Water samples were collected at a depth of 0.5 m, stored in 1L polyethylene bottles and kept in coll boxes until arrival to the laboratory, where they were stored at 4 °C.

Sediment samples were taken from the reaches where sediment deposition occurs. Usually a few meters from the riverbank. Wherever possible, 3 kg of the 15 cm sediment layer was collected into polyethylene containers and transported to the laboratory. In the laboratory, sediment samples were homogenized and wet sieved through a 63 μm sieve [42,43]. After drying at 40 °C to a constant weight, the samples were kept in polypropylene containers in the dark at 4 °C. The moisture content was determined by drying the sediment at 60 °C until constant weight. All analyses were done in three replications, and the results were calculated on a dry-mass basis.

### 2.3 Determination of total element concentrations in water and sediments

Concentrations of the elements (As, Cu, Cd, Cr, Pb, Zn, Ni and Fe) were determined in the sediment and water samples. To determine the soluble PTE concentrations, water samples were filtered before analysis through 0.45 μm filters cellulose nitrate membrane (Sartorius, Goetingen, Germany) (25 mm diameter). Water samples were acidified with 1 mL of suprapure HNO_3_ per 1 L of sample. For determination of element concentration in sediments, approximately 0.25 g of dry sediment sample was weighed into a Teflon tube and subjected to microwave-assisted digestion using a mixture of nitric, hydrofluoric, hydrochloric and boric acid [44]. A CEM Corporation (Matthews, NC, USA) CEM MARS 5 Microwave Acceleration Reaction System was used for digestion of the sediments. Element concentrations in the water and digested sediment samples were determined by inductively coupled plasma mass spectrometry (ICP-MS) on an Agilent Technologies (Tokyo, Japan) 7700x ICP-MS.

### 2.4 Quality control of the analytical methods

To check the accuracy of the determination of total element concentrations in water samples, SPS-SW1 (Quality Control Material for Surface Water Analysis) was analyzed, while the certified reference material CRM 320R (Trace Elements in Chanel Sediment) was analyzed for the total element determination in sediments. The measured values varied from 96 % to 103 % which indicates good agreement between the determined and certified values, which confirms the accuracy of the analytical methods used.

### 2.5 Statistical analyses

The distribution patterns of elements in water and sediment samples along the Sava River were assessed using linear discriminant analysis (LDA) [45]. Alpha diversity was calculated using the Shannon Weaver function:

(2) $$H=-\sum_{i=1}^{s}p_{i}\;\log\;p_{i}$$

where p_i_ is the proportion for ith species within a community, and s the number of species within the community [46]. Equitability was assessed using the relation [47]:

(3) $$E=\frac{H}{H_{\max}}=\frac{-\sum_{i=1}^{s}p_{i}\;\log\;p_{i}}{\log\;s}$$

Beta diversity was calculated according to:

(4) $$\beta =\frac{l_{\left(H\right)}+g_{\left(H\right)}}{2}$$

where l_(H)_ is number of species lost, and g_(H)_ the number of species gained along the habitat gradient [48].

We assessed the mutual relationship among aquatic vegetation and water and sediment chemistry using both ordinary and partial Mantel tests [[49], [50], [51]]. To obtain more details of the relationship between the distribution of each species and the concentration of each element in the sediments and water, we performed canonical correspondence analysis [52].

The response of macrophyte communities to an increased level of PTEs was investigated using nonlinear regression analyses, based on either Gaussian or beta distribution models [53]. All statistical analyses were performed using the “FLORA” software package [54].

## 3 Results and discussion

### 3.1 Sediment and water chemistry

The range of the soluble concentrations of the selected elements (As, Cu, Cd, Cr, Pb, Zn, Ni, and Fe) in the filtered water samples are shown in Table 2. The results are compared with the global average values of total dissolved elements in natural river systems (large rivers worldwide excluding polluted rivers). A comparison is also made with the environmental quality standards for dissolved elements set out in the Water Framework Directive [[55], [56], [57], [58]]. The results showed that the content of As, Cd, Cr, and Fe in the water at all the localities was significantly lower in relation to the world average, while the content of Cu, Ni, Pb and Zn in individual localities had a value greater than the world average levels, but lower than the annual average given by the WFD. In respect to elements regulated by the environmental quality standards [58], the concentration of Fe exceeded annual average values at sites Slavonski Brod, Županja, and Belgrade. Concentrations of Cd were below the limit of quantification (LoQ).

**Table 2:** The range of concentration of selected elements in filtered water samples and data on sites with exceeding values.

Sampling site	Elements (μg L^−1^)							
	As	Cd	Cr	Cu	Ni	Pb	Zn	Fe
Min-Max	0.142–1.910	<LoQ	0.113–0.474	0.220–2.240	0.212–13.400	0.021–0.265	<LoQ-6.980	0.307–29.400
Above world average	No exceeding	No exceeding	No exceeding	RAD, BEO	All, except: MOJ, RAD, CAT, ZAG, JAS	LIT1, LIT2, VRH, ZAG	All except: MOJ, ZUP, SRM1	No exceeding
Above AA	–	No sites	No sites	No sites	No sites	No sites	No sites	SLB,ZUP, BEO
World average^55^	0.11–9.4^59^	0.08	0.7	1.48	0.8	0.079	0.6	66
AA(EQS)^58^	–	≤0.08	3.4–4.7	5	20	7.2	8	16^57^

^55^Gallardet et al., 2004; ^58^Annual Average Environmental Quality Standards set by Water Framework Directive (WFD 2009); ^59^Kabata-Pendias and Mukherjee, 2007; ^57^Crane et al., 2007.

**Fig. 2:**
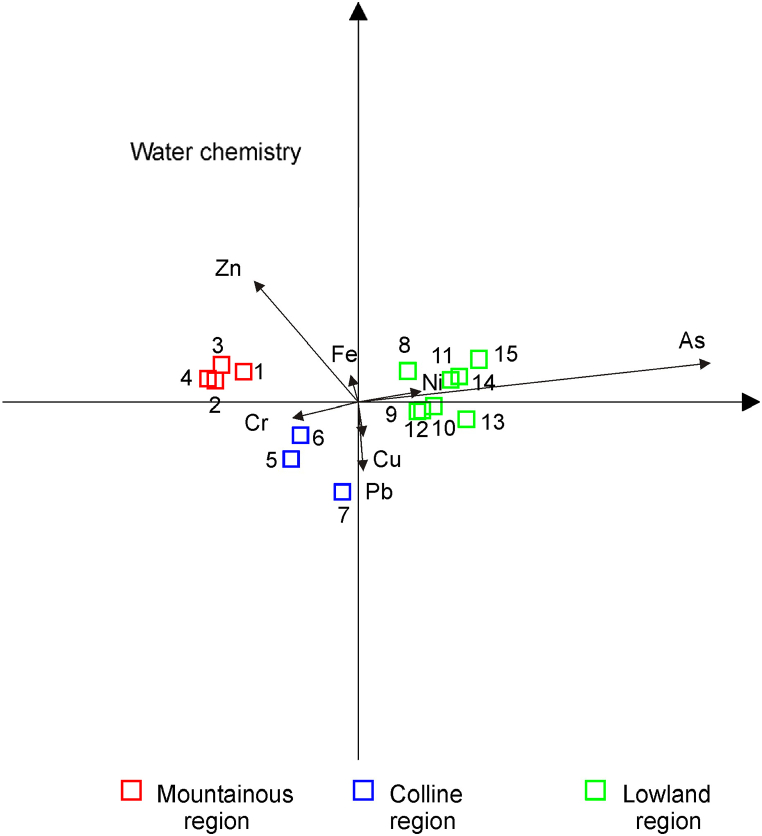
Distribution of elements in water samples (Sampling sites labelled 1–15, see Table 1).

The concentration of As gradually increases along the watercourse. The highest concentration was recorded in Belgrade (1.91 μg L^−1^). A similar distribution was observed for Ni. Quite the opposite trend was recorded for Cr, Pb, and Zn, the highest concentrations of which were detected in upstream sites. Such a pattern may be explained by the natural geochemical weathering of minerals, but also by anthropogenic sources [21,22,59]. Concentrations of Cu and Fe had an irregular and multimodal distribution. On the basis of the obtained results, it is evident that the Sava River is under anthropogenic influence. In order to find the linear combinations of the analyzed elements that maximally discriminate the analyzed sampling sites linear discriminant analysis (LDA) was used. The LDA results clearly separate sites from the upper, middle, and lower course, along the As and Ni concentration gradients (Fig. 2).

**Fig. 3:**
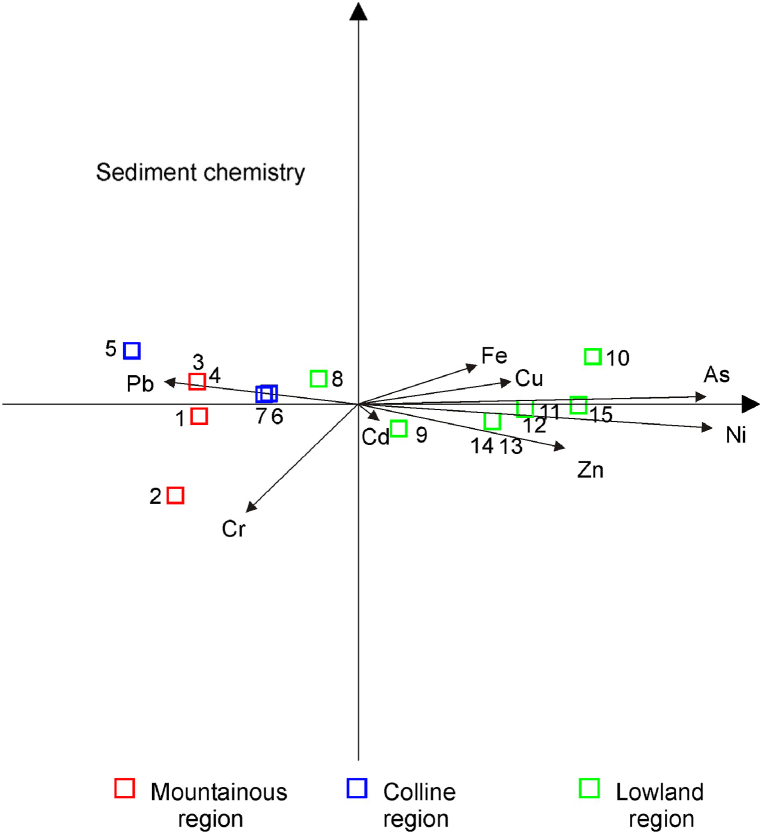
Distribution of PTEs in sediments (Sampling sites labelled 1–15, see Table 1).

To estimate the environmental status of sediments of the Sava River, the Canadian Environmental Quality Guidelines for the Protection of Aquatic Life [60] were used. In that respect, Interim sediment quality guidelines (ISQG) regulated by this standard are recommended for total concentrations of chemicals in freshwater surficial sediments (upper layer, app. 5 cm), as quantified by standardized analytical protocols for each chemical. In comparison to water, the sediment chemistry shows a slightly different pattern (Table 3). Higher concentrations (above ISQG) of As, Cr, Pb, and Zn were recorded at the upper-stream sites and also at the downstream sites (especially Sremska Mitrovica, Šabac, and Belgrade), whereas the Cd content in the sediment at the Mojstrana site was equal to the ISQG level. In general, the content of all examined elements in sediments of the Sava River was lower than the probable effect level (PEL) values, apart from Cr. Concentrations of As and Ni gradually increase along the watercourse and together with Cr reach their highest values in the downstream part of the Sava River. Similar results were reported earlier by Milačič et al. [24], who found a moderate elevation of Cr and Ni (up to 400 and 210 mg kg^−1^, respectively) in the sediment. Compared to water chemistry, sediment chemistry better separates the sites (Fig. 3).

**Table 3:** Total concentrations of selected elements in sediments.

Sampling site	Elements (mg/kg; for Fe 10^3^ mg/kg)							
	As	Cd	Cr	Cu	Ni	Pb	Zn	Fe
Min-Max	4.01–13.70	0.22–0.68	23.80–191.00	8.14–32.00	9.64–104.00	18.90–51.80	31.10–172.00	6.00–33.10
Above ISQG	RAD, VRH, SLB, ZUP, SRM2, SAB1, BEO	MOJ	All except: MOJ, LIT2, ZAG	No sites	–	RAD, VRH, SRM2	RAD, SRM2	–
Above PEL	No sites	No sites	RAD, ZUP, SRM2 SAB1	No sites	–	No sites	No sites	–
ISQG^a^	5.9	0.6	37.3	35.7	–	35.0	123.0	–
PEL^a^	17.0	3.5	90.0	197.0	–	91.3	315.0	–

a Canadian Environmental Quality Guidelines [60]; ISQG − corresponds to the threshold level effects below which diverse biological effects are not expected; PEL − concentrations of pollutants that may affect the aquatic life.

If we compare the contamination detected herein with those in similar water types, the concentration of As, Cd, Cu, Pb, and Zn in the sediments of the Sava River was in general lower than those found in the Danube River [13,43] and Tisa River [42]. The higher concentrations of Cr and Ni in the Sava, in comparison to their concentrations in the Tisa and Danube, were an exception. In view of the geological background, increased levels of these elements are to be expected in the lower reaches of the river. Namely, the central Dinaric ophiolite belt [61] and the serpentinized alluvial deposits of Kolubara from the Maljen and Rudnik Mountains [62] are present in the lower reaches of the Sava. The levels of these elements in the sediment of the Sava River also exceeded the limits proposed for PTEs in the sediment of the Danube River basin.

### 3.2 Flora of macrophytes

Floristic research at 15 selected sites along the River Sava established the presence of 26 macrophytic species grouped in 17 plant families (Table 4). Among the recorded species, we identified one from the green algae division (Chlorophyta: *Cladophora* sp.), two from the moss division (Bryophyta: *Cinclidotus fontinaloides* and *Fontinalis antipyretica)*, the species *Salvinia natans*, which is an annual floating aquatic fern (division Polypodiophyta), and 22 species of angiosperms. Among the angiosperms, the richest in species is the *Potamogetonaceae* family (6 plant species), followed by *Hydrocharitaceae* (3 species), while 2 species were recorded in each of the families *Ceratophyllaceae* and *Araceae*. The highest occurrence frequency was that of the filamentous alga *Cladophora* sp. that appeared in 11 (73.33 %) of the localities, while other species had significantly lower frequencies of occurrence (*Myriophyllum spicatum -* 46.66 %; *Bolboschoenus maritimus -* 40 %). *Ceratophyllum submersum, Lemna minor, C. fontinaloides, Najas marina, Potamogeton crispus, Stuckenia pectinata* and *S. natans* were found at 5 (33.33 %) of the sampling sites. Specific to the mountainous and colline parts of the River Sava are *Ranunculus trichophyllus* and the mosses *C. fontinaloides* and *F. antipyretica*, while *M. spicatum* has the highest abundance. Localities in the lowlands of the Sava have a considerably higher abundance and frequency of occurrence of macrophytic species. On these sites alone there are 18 species (69.2 %), and the most frequent are *B. maritimus*, *C. submersum, Cladophora* sp., *L. minor*, *N. marina*, *P. crispus*, *S. natans* and *Trapa natans*. In the locality Belgrade, the highest number (5) of the species *L*. *minor* and *C*. *submersum* was recorded, and these were also the most numerous in the Šabac – upstream locality. There is a slightly lower abundance (4) of *Ceratophyllum demersum* (SRM1), *C. submersum* (SLB, SRM1*), Cladophora* sp. (SAB1), *N. marina* (SAB1) and *Sagittaria sagittifolia* (SRM2, SAB1).

**Table 4:** Macrophyte species recorded at sampling sites.

Plant species	Abbr.	Sampling sites														
		1	2	3	4	5	6	7	8	9	10	11	12	13	14	15
*Bolboschoenus maritimus* (L.) Palla (Cyperaceae)	Bol mar	.	.	.	.	.	.	.	3	2	2	2	2	3	.	.
*Butomus umbellatus* L. (Butomaceae)	But umb	.	.	.	.	.	.	.	.	.	.	2	2	3	.	.
*Ceratophyllum demersum* L. (Ceratophyllaceae)	Cer dem	.	.	.	.	.	.	.	.	.	3	4	.	.	.	.
*Ceratophyllum submersum* L. (Ceratophyllaceae)	Cer sub	.	.	.	.	.	.	.	.	4	.	4	3	5	.	5
*Cinclidotus fontinaloides* (Hedw.) P.Beauv. (Pottiaceae)	Cin fon	2	1	1	2	2	.	.	.	.	.	.	.	.	.	.
*Cladophora* sp. (Cladophoraceae)	Cla sp	2	2	.	3	.	.	2	2	3	2	2	3	4	1	
*Fontinalis antipyretica* Hedw. (Fontinalaceae)	Font ant	2	.	2	2	2	.	.	.	.	.	.	.	.	.	.
*Elodea canadensis* Michx. (Hydrocharitaceae)	Elo can	.	.	.	.	.	.	.	.	.	.	.	.	3	.	.
*Iris pseudoacorus* L. (Iridaceae)	Iri pse	.	.	.	.	.	.	.	.	.	.	.	.	1	2	.
*Isöetes sp.* (Isoetaceae)	Iso sp	.	.	.	.	.	.	.	.	.	.	.	.	.	1	1
*Lemna minor* L. (Araceae)	Lem min	.	.	.	.	.	.	.	2	2	2	.	.	2	.	5
*Myriophyllum spicatum* L. (Haloragaceae)	Myr spi	.	3	2	3	3	3	3	.	.	.	.	.	2	.	.
*Najas marina* L. (Hydrocharitaceae)	Naj mar	.	.	.	.	.	.	.	.	3	2	3	2	4	.	.
*Nuphar luteum* (L.) Sm. (Nympheacee)	Nup lut	.	.	.	.	.	.	.	.	3	.	.	2	1	.	.
*Potamogeton crispus* L. (Potamogetonaceae)	Pot cri	.	.	.	.	.	.	.	.	3	.	3	3	3	.	2
*Potamogeton fluitans* Roth (Potamogetonaceae)	Pot flu	.	.	.	.	.	.	.	.	2	.	.	.	2	.	1
*Potamogeton gramineus* L. (Potamogetonaceae)	Pot gra	.	.	.	.	.	.	.	.	.	.	3	.	3	.	.
*Potamogeton lucens* L. (Potamogetonaceae)	Pot luc	.	.	.	.	.	.	.	.	2	2	.	.	.	1	.
*Stuckenia pectinata* (L.) Börner (Potamogetonaceae)	Pot pec	.	.	.	3	.	.	.	.	2	.	3	3	3	.	.
*Potamogeton perfoliatus* L. (Potamogetonaceae)	Pot per	.	.	.	2	.	.	.	.	4	.	.	.	.	.	.
*Ranunculus trichophyllus* Chaix (Ranunculaceae)	Ran tri	.	3	2	.	.	.	.	.	.	.	.	.	.	.	.
*Sagittaria sagittifolia* L. (Alismataceae)	Sag sag	.	.	.	.	.	.	.	.	2	.	.	4	4	1	.
*Salvinia natans* (L.) All. (Salviniaceae)	Sal nat	.	.	.	.	.	.	.	2	2	2	.	.	1	.	3
*Spirodela polyrhiza* (L.) Schleid. (Araceae)	Spi pol	.	.	.	.	.	.	.	.	.	.	.	.	.	1	3
*Trapa natans* L. (Lythraceae)	Tra nat	.	.	.	.	.	.	.	2	2	.	.	2	3	.	1
*Vallisneria spiralis* L. (Hydrocharitaceae)	Val spi	.	.	.	.	.	.	.	.	.	2	.	.	1	.	.

Sampling sites: 1. MOJ; 2. RAD; 3. LIT1.; 4. LIT2.; 5. VRH; 6. CAT; 7. ZAG; 8. JAS; 9. SLB; 10. ZUP; 11. SRM1; 12. SRM2; 13. SAB1; 14. SAB2; 15. BEO.

In comparing the macrophytic species recorded in the River Sava with the species in other rivers, we found significant similarities. An example of a tributary of the Sava is the Ljubljanica River in Slovenia. This karstic river system consists of seven interconnected surface waters, both permanent and intermittent, which are linked by underground groundwater flows. In the area of the Ljubljanica river, of the total 62 macrophytic taxa identified, 14 were also found in the River Sava (Cs = 31.8 %). In addition, in the Krka River (Slovenia), alongside the high diversity of macrophytes (39 species), a large abundance of the submersed species *M. spicatum* and *N. marina* was recorded, similar to the Sava [63]. The Sørensen similarity index of the macrophytic flora of these two rivers is 43.1 %. It was established that *Elodea canadensis* prevailed in shallower, calm locations near the riverbank of the Krka, as it does in a similar locality in Šabac (SAB1), where it was also recorded. In the Zasavica, a slow-flowing lowland river found in the Special Nature Reserve “Zasavica” and which is connected to the Sava River, 29 species of macrophytes were recorded (Cs = 40 %). The macrophyte vegetation of the international River Danube comprises 51 species, of which 18 are also found in the River Sava (Cs = 46.75 %). The mosses *C. fontinaloides* and *F. antipyretica* have the highest occurrence frequency in the upper course of these rivers, which confirms the fact that the submersed bryophytes of temperate regions are typical dominant plants in habitats presenting fast-flowing waters [[64], [65], [66]]. At the other extreme of the flow gradient of the rivers Sava and Danube, *Potamogeton nodosus* (sin *P. fluitans*) and the pleustophyte *L. minor*, as the most significant indicator species for low-flow velocities, were detected [67]. Comparison of the macrophytic flora of the Sava and Tamiš (Serbia) rivers showed a significant similarity, which was confirmed by the Sørensen similarity index (52.63 %). The highest diversity of macrophytic flora was recorded in the plain section of the Tamiš, similar to the River Sava [68]. The presence of the submersed species *E. canadensis* and *Vallisneria spiralis*, which are numerous in submersed vegetation and the result of the introduction of invasive species that out compete native plants, is a characteristic of both rivers [69].

### 3.3 Diversity of macrophyte communities

Biodiversity (biotic variability) may be classified using different approaches [70]. According to Whittaker [71], the diversity of biotic communities is divided into alpha, beta and gamma components. Alpha diversity (within-community diversity) depends on species richness (number of species within a community) and dominance of species (proportion of individuals of a particular species with respect to individuals of all species within a community). Dominance of species is frequently referred to as “species equitability”. Beta diversity (diversity between communities) may be divided into directional turnover along a gradient and non-directional variation among communities [72]. Directional beta diversity quantifies the change in community composition along a spatial, temporal or environmental gradient, observing how communities change between sampling units. In contrast, undirected beta diversity reflects the overall variation in community composition across all possible pairwise comparisons of sampling units, without considering a specific environmental gradient.

**Fig. 4:**
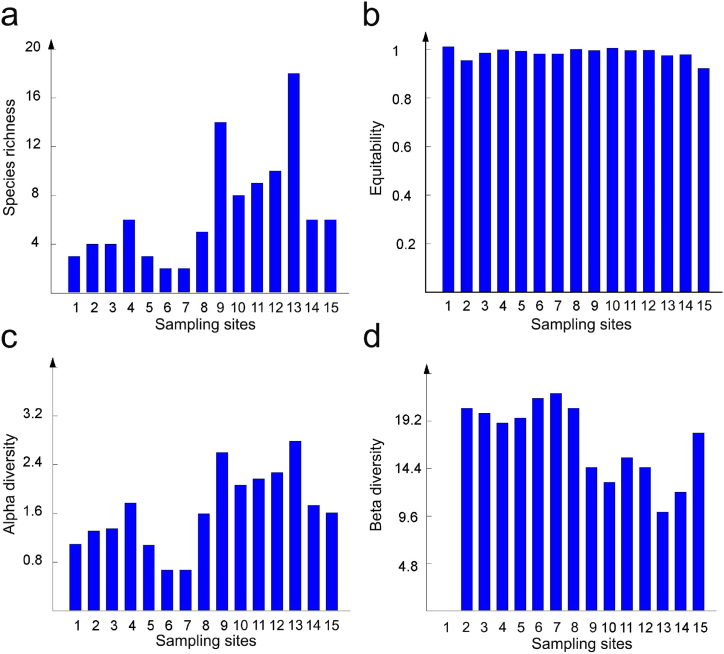
Diversity components of macrophyte communities along the Sava River: a) species richness; b) equitability; c) Alpha diversity; d) Beta diversity (Sampling sites labelled 1–15, see Table 1).

The different biodiversity components of macrophyte vegetation along the Sava River are presented in Fig. 4.

The low number of species was recorded in upper-stream sites, which may be explained by unfavorable physical conditions (high flow velocity, torrential fluctuations of water level, type of sediment) [14,73]. Fast water flow is a powerful selective pressure, since fluctuating water levels can mechanically disturb the stream bottom and have disastrous effects on macrophyte populations [74]. Therefore, only a few macrophyte species, mainly mosses, may persist in such a harmful habitat.

In the present study, the species richness of macrophyte communities generally increases towards downstream sites (Fig. 4a). Such a trend may be explained by the change in sediment type (increase of silty substrate favorable for rooting macrophytes) [75]. The highest number of species was recorded in the upstream Šabac site. However, such a trend is interrupted in hilly localities (VRH, CAT, and ZAG). The decrease in diversity at these sites is due to the absence of moss species characteristic for the upper stretch of the river. The general change of river type that includes the substrate type, water hydrology, and chemistry, does not favour the development of moss communities. At the same time, the stretch between Vrhovo and Zagreb does not provide conditions for the growth of typical lowland macrophyte vegetation, mostly because of the absence of a soft sediment which is required for rooting. A reduction in species richness was also recorded at the downstream sites Šabac and Belgrade, where it could be attributed mostly to anthropogenic pressures. These sites are under the significant influence of nearby industries (textile, pharmaceutics, fertilizers, paper production, food industry, as well as pollution from thermal power plants and related large ash disposal sites). Human activity leads to a loss in biodiversity and impaired ecosystem functioning [76]. Eutrophication, pollution with PTEs, enrichment of water bodies with phosphate derived from agriculture, sewage and industry are considered to be the key drivers of aquatic plant loss [76,77].

Variations in the trends of species richness and alpha diversity are similar (Fig. 4a–c). The equitability component of alpha diversity approaches to 1 in all of the analyzed sites, indicating that macrophyte communities along the Sava River exist in a less disturbed environment [78]. Compared to alpha diversity, beta diversity has quite an opposite trend (Fig. 4c and d). The greatest species turnover was recorded in the upper-stream sites. Environmental conditions (river depth and width, type of sediment, water velocity, temperature, turbidity, conductivity, etc.) vary widely in upper-stream sites, affecting fast species replacement and the diversification of aquatic vegetation [79]. Peaks of beta diversity were recorded in the Čatež and Zagreb sites. Most of the moss macrophytes recorded in upper-stream sites disappear in the colline area, causing an increase in beta diversity. Generally, moss species are characteristic for the upstream parts of rivers with higher flow velocity [80,81].

Downstream of Zagreb, the beta diversity successively decreases. This situation is attributable to the increased similarity of environmental conditions in the downstream sites. Similar environmental conditions result in a similar floristic composition of aquatic communities. A sharp beta diversity change was observed in Šabac and Belgrade, which is not a consequence of increased community diversification, but rather the significant degradation of aquatic communities. Many species common to lowland sites disappear in Šabac and Belgrade (Table 4). Species reduction may be attributable to increased anthropogenic pollution. Sediments in these two sites are characterized by increased concentrations of PTEs, especially Cr (above the PEL values) (Table 3).

### 3.4 Relationship between macrophyte communities and sediment and water chemistry

In order to investigate the mutual relationship between macrophyte communities and water and sediment chemistry, we performed ordinary and partial Mantel tests to detect the significance of the null hypotheses that concentrations of elements either in water or in sediment samples are uncorrelated with macrophyte communities. Results of ordinary and partial Mantel tests are presented in Table 5.

**Table 5:** Mantel test results; * for p < 0.05

Type of correlation	Ordinary Mantel correlation	Partial Mantel correlation
Macrophytes/Water chemistry	r = 0.311 p = 0.085	r = 0.060 p = 0.804
Macrophytes/Sediment chemistry	r = 0.387 p = 0.016*	r = 0.351 p = 0.026*

Statisticaly significant correlations are denoted in bold.

Ordinary Mantel correlation coefficients point to a statistically significant relation between sediment chemistry and macrophyte communities. However, macrophyte communities are poorly related to water chemistry, since the probability of the hypothesis that elements in water are unrelated with macrophyte communities is much above the significance level of 5 %. Such a result may be explained by the fact that the concentrations of the examined elements in the water were lower than the average values of these elements [58]. Trace element accumulation in sediments is the result of long-term exposure, whereas trace element concentrations in water are mainly the result of recent contamination [82]. The sediment chemistry is important for many rooting macrophytes, because the surface of sediment is the most important reservoir or sink of trace elements and other pollutants in aquatic environments [59,83]. Moreover, compared to the highly fluctuating and ephemeral concentrations of elements in water, the sediment chemistry is more stable. Due to adaptations to relatively stable environmental conditions, the macrophyte communities are correlated to sediment chemistry.

**Fig. 5:**
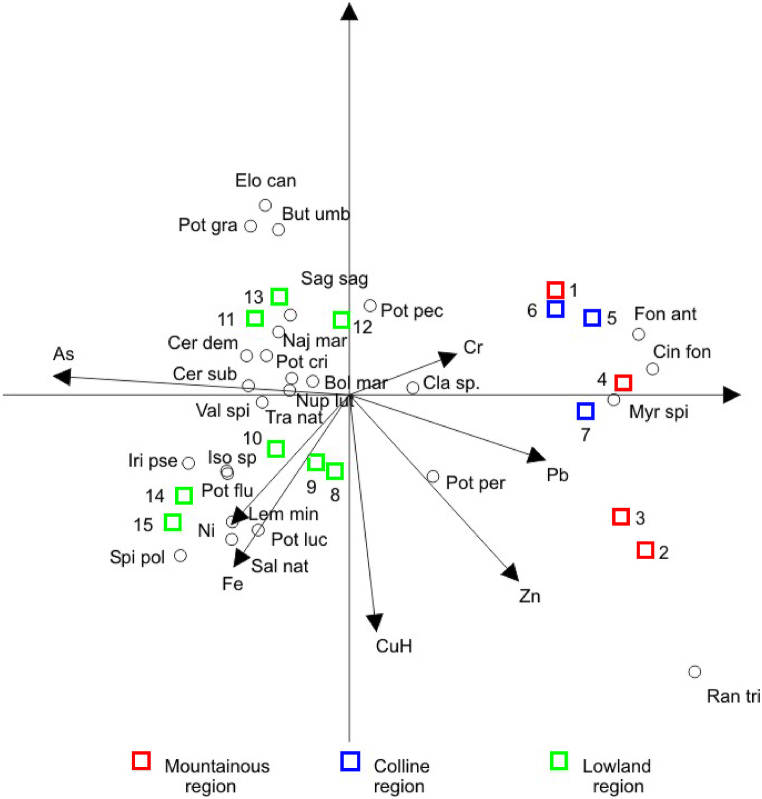
Relationship between water chemistry and macrophyte communities along the Sava River (Sampling sites labelled 1–15, see Table 1).

The effects of water chemistry on the distribution of macrophyte species is presented in Fig. 5. A strong gradient of As along the Sava River separates the upper- and downstream sites. On the other hand, downstream sites form a separate gradient along the second axis. The most important elements separating the lowland sites are Ni and Fe, which increase towards Belgrade and Šabac. The numbers of *Spirodela polyrhiza, L. minor, Potamogeton lucens* and *S. natans* in these sites are significant. *M. spicatum, Potamogeton perfoliatus* and the moss species *F. antipyretica, C. fontinaloides* dominate in the upper-stream sites, where a high concentration of Pb and Cr was detected.

**Fig. 6:**
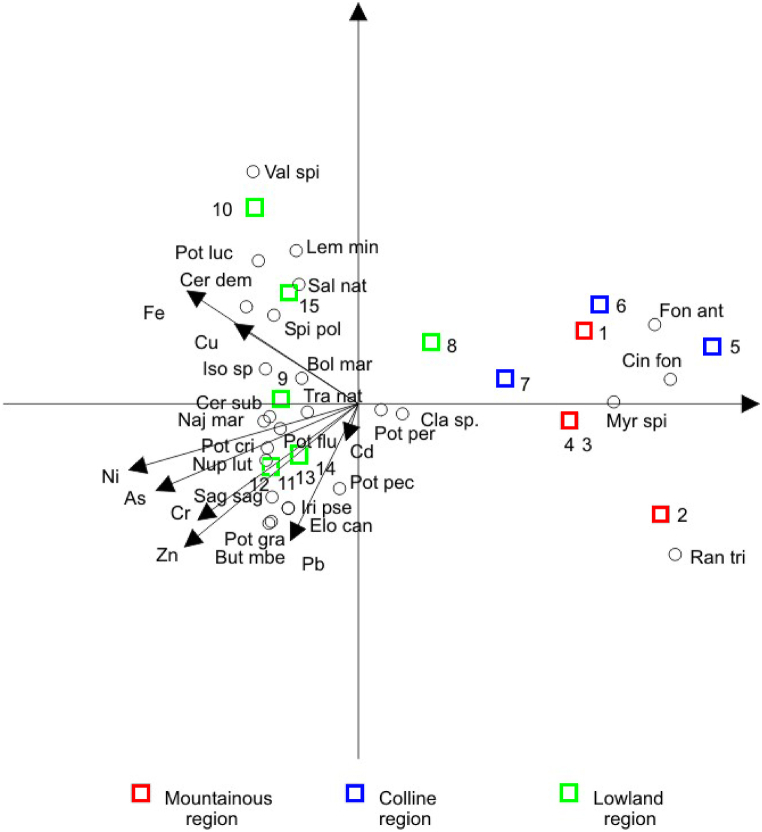
Relationship between sediment chemistry and macrophyte communities along the Sava River (Sampling sites labelled 1–15, see Table 1).

The correlation of sediment chemistry on the distribution of macrophytes is presented in Fig. 6. Canonical correspondence analysis specifies the linear combination of elements in sediment samples that maximizes the dispersion of the species, thus indicating that most species that are distributed in the lowland sites are adapteded to the increased concentrations of As, Cr and Ni in the sediment.

### 3.5 Impact of PTEs on macrophyte communities

**Fig. 7:**
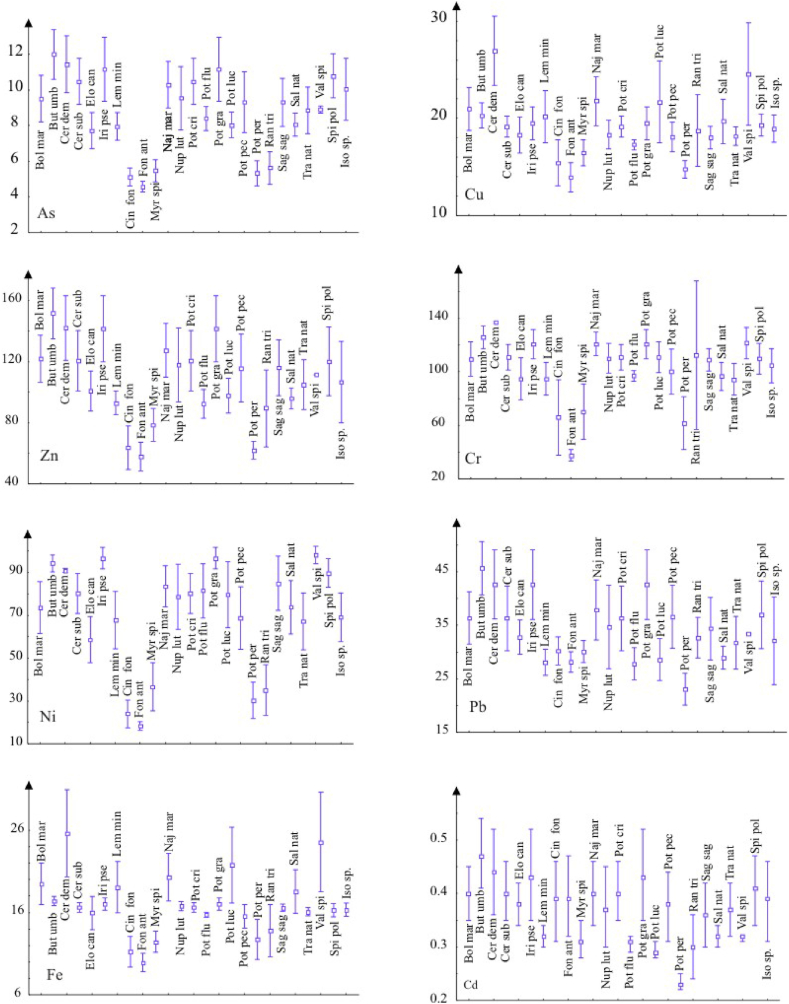
Distribution of macrophyte species with respect to concentrations of elements in sediment samples (y axes = concentration of elements in mg kg^−1^. Each label on x axis specifies particular species).

An excessive load of chemical elements in aquatic communities could lead to the disappearance of sensitive species [84]. Macrophyte communities are poorly correlated with water chemistry (Table 5). Therefore, we analyzed the distribution of the macrophyte species with respect to the concentration of elements in sediment only (Fig. 7).

*Butomus umbellatus, C. demersum, C. submersum, Iris pseudacorus* and *Potamogeton gramineus* are tolerant to increased concentrations of As. *C. demersum, P. lucens* and *V. spiralis* have a wide ecological amplitude with respect to concentrations of Cu in sediment. Other species are much more sensitive to increasing Cu content. *B. umbellatus, C. demersum, I*. *pseudacorus* and *P*. *gramineus* inhabit sites with increased Zn concentration. *P*. *gramineus, C*. *demersum, N*. *marina, B. umbellatus, V*. *spiralis* and *S*. *polyrhiza* tolerate sites with increased Cr concentrations. The most tolerant species with respect to increased Ni concentrations are *V*. *spiralis, S*. *polyrhiza, P*. *gramineus, C*. *demersum, B. umbellatus* and *I*. *pseudoacorus*. *C*. *demersum, P*. *lucens* and *V*. *spiralis* have a wide ecological amplitude with respect to concentrations of Fe in sediments. In addition, *B. umbellatus, C*. *demersum, I*. *pseudacorus* and *P*. *gramineus* have a wide ecological amplitude with respect to concentrations of Cd in sediments. Analysis revealed a group of species known to be comparatively more tolerant to high concentrations of PTEs; these are: *C*. *demersum, I*. *pseudacorus, N*. *marina, B*.*umbelatus, V*. *spiralis, P*. *gramineus* and *B*. *maritimus*. These species could be used in phytoremediation.

*P*. *perfoliatus*, as well as moss species *C*. *fontinaloides* and *F*. *antipyretica*, have narrow ecological amplitudes with respect to the concentrations of PTEs in the sediment. *F*. *antipyretica, C*. *fontinaloides* i *P*. *perfoliatus* are sensitive to increased concentrations of most of the examined elements, with *F. antipyretica* being sensitive to increased concentrations of As, Cu, Zn, Cr, Ni, Pb, Fe, *C. fontinaloides* to As,Zn, Ni, Pb and Fe, and *P. perfoliatus* to As, Cu, Zn, Ni, Pb and Cd. Thus, our data suggest that these stenovalent species can serve as indicators of PTEs in sediment. However, additional studies are required to select appropriate indicators and community metrics effective for the assessment of the status of water bodies which also include the study of bio-accumulation in plant tissues. The size of the sample also has to be considered in order to obtain an effective assessment (in terms of confidence and economic effectiveness).

**Fig. 8:**
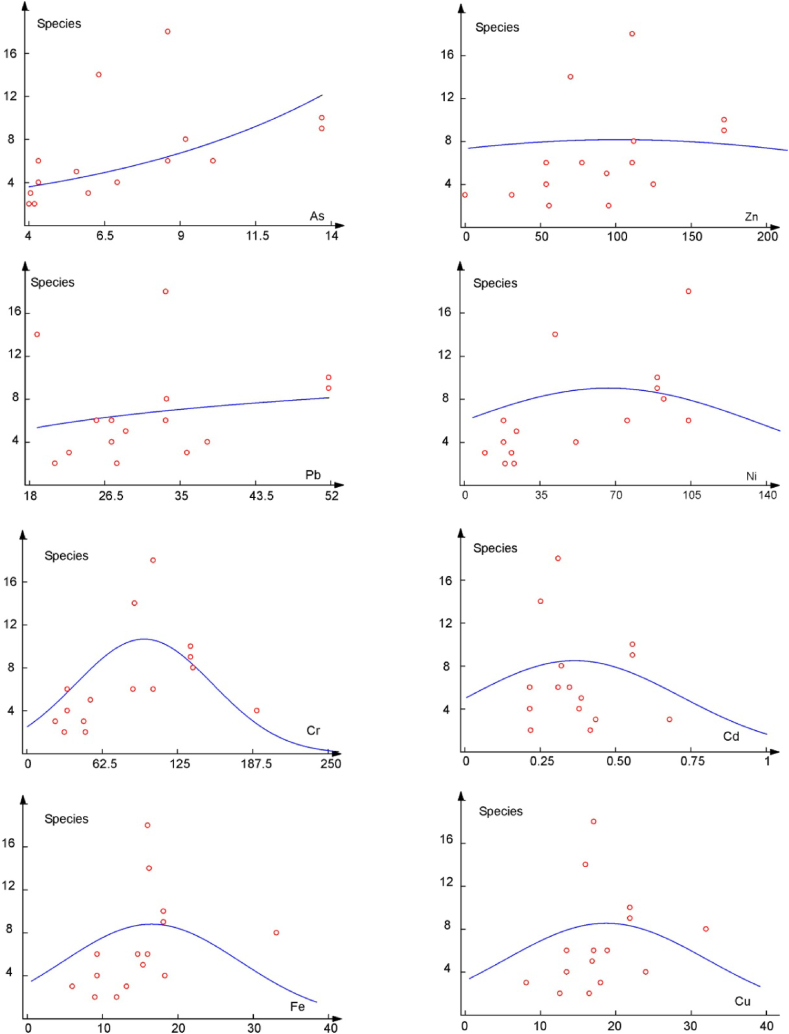
Relationship between the species richness of macrophyte communities and concentrations of elements in sediment along the Sava River (x-axis = concentration of elements in mg kgL^−1^; y-axis = species richness).

Gaussian and beta distributions may be used for the modeling of species response to spatial, temporal or environmental gradients [53,[85], [86], [87], [88]]. We used these distributions to investigate the response of macrophyte communities (expressed in terms of species richness) to an increased level of the analyzed chemical elements in sediment (Fig. 8).

Species richness increases along the gradient of As concentrations in sediment (Fig. 8). However, the increase of species richness could not be explained by the As concentration, but by the diversification of habitat conditions and increase of silty substrate favorable for rooting macrophytes. Thus, a positive correlation between species richness and As content is a side effect (Fig. 6, Fig. 7, Fig. 8). Species richness is poorly correlated with concentration gradients of Pb, Zn and Ni.

Species richness changes in response to concentration gradients of Cr, Cd, Fe, and Cu. Thus, species richness is relatively low at sites with low concentrations of these elements in the sediment, and increases with increasing concentrations of elements, albeit only up to a certain level. A further increase in Cr Cu, Cd, and Fe concentrations in sediment samples decreases species richness, probably due to increased toxicity (Fig. 8). Our data indicate that a decrease of species richness occurs above the following concentrations of Cu > 17.5 mg kg^−1^, Cd > 0.3 mg kg^−1^, Cr > 102 mg kg^−1^ and Fe > 16 mg kg^−1^. Cu and Fe are essential nutrients, and deficiencies of these elements can cause significant metabolic disorders [89]. Although Cr is not an essential element, in small quantities it can have a positive effect on plant growth, while in larger concentrations it can have very toxic inhibitory effects on plant growth and development [90,89]. Considering that the highest concentrations of Cr in the sediment were recorded downstream, in Radovljica, Županja, and Sremska Mitrovica downstream, suggests that the macrophytes found at these localities are tolerant to increased concentrations of Cr (>PEL values; Table 3).

The considerable diversity and distribution of biota along the Sava is due to the heterogeneity of environmental conditions. These include variations in the hydrological regime, climatic factors, geological composition (petrography), soil characteristics (pedology) and topographical features. The Sava River comprises a series of flowing water bodies of varying turbidity and depth, as well as slower-flowing streams and lenticular habitats. In addition, the landscape consists of a mosaic of flat or slightly hilly terrain interspersed with meanders, side channels, oxbow lakes, islands and ridges. These diverse environmental conditions together contribute to the great biodiversity along the Sava River [91]. All this affects the diversity of riverine, lacustrine and palustrine communities that are exposed to increasing pressure from a multiple environmental stressors [14,92].

The results obtained in this study indicate that the concentration of the analyzed chemical elements in sediment increases along the Sava River in a downstream direction. Local concentration peaks be attributable to both natural processes (leaching and geological weathering processes) and anthropogenic impacts (industrial and communal discharge, agricultural diffuse contamination sources, mining, etc.). Due to their high toxicity, persistence and tendency to accumulate in sediment and living organisms, PTEs present one of the major contamination problems for freshwater systems [21]. The level of PTE bioaccumulation in the tissues increases with the increasing trophic level of aquatic organisms [93]. A widely accepted model describing the impact of pollution on communities is based on the assumption that increased pollutant load and decreased diversity are correlated. Low diversity is a consequence of the high dominance of a limited number of opportunistic species capable of reaching high densities in adverse environmental conditions and by the recession of a number of species intolerant to such conditions [94,95].

However, the results of this study are in disagreement with the assumptions of the model. The equitability component of alpha diversity approaches 1 at all of the analyzed sites (Fig. 4b). Supra-optimal concentrations of PTEs affect the growth and development of some PTE-sensitive macrophytes along the Sava River. Species resistant to high levels of PTEs either avoid excessive uptake of toxic elements or tolerate an excess of harmful elements by utilizing different detoxication mechanisms [[96], [97], [98]].

As there was mentioned before, macrophyte communities along the Sava River are significantly correlated with the concentration gradients of elements in sediment. Our results confirm that besides the clear correlation of the sediment determinants examined in the study with species richness and diversity metrics, it is difficult to obtain a clear conclusion about the direct relationships between aquatic biota and contaminants, as communities, including plants, are strongly dependent on environmental factors related to water body type – current velocity, substrate type and other related variables, which has been established in a number of studies [14,[99], [100], [101], [102]].

Analysis of the concentrations of trace elements in plant tissues aimed at establishing the actual impact of individual elements on species richness along the River Sava will be the subject of further research.

## 4 Conclusions

In the present study, the floristic composition of macrophytes and their distribution along the Sava River, as well as the potential effects of As, Cu, Cd, Cr, Pb, Zn, Ni and Fe in the water and sediment on macrophyte communities were analyzed.

Macrophyte communities, which included 26 plant species classified into 17 families, significantly correlated with sediment chemistry, but poorly correlated with water chemistry. Gradual increases in the concentrations of the analyzed elements in the sediment along the Sava River in the downstream direction were observed. Both species richness and alpha diversity of macrophyte communities generally increased in the downstream direction. However, this trend is interrupted at several localities (VRH, CAT and ZAG in upper and SAB2 and BEO in the lower stretch). In the upper stretch, the decrease in diversity is due to the absence of moss species characteristic for the upper stretch of the river and because of the overall change in river type, whereas the lower species richness in the lower stretch can mostly be attributed to anthropogenic pressures.

The equitability component of alpha diversity for the macrophyte community approaches unity at all of the analyzed sites, pointing to a good environmental status. In lowland regions (downstream), the beta diversity decreases. This can be attributed to an increased similarity of environmental conditions at downstream sites. Similar environmental conditions give rise to a similar floristic composition of aquatic communities.

Analysis of distribution of macrophyte species with respect to different concentrations of elements in the sediments revealed a group of species that was generally more tolerant to high concentrations of PTEs; these are: *C*. *demersum, I*. *pseudacorus, N. marina, B*.*umbelatus, V*. *spiralis, P*. *gramineus* and *B*. *maritimus*. These species can be potentially useful in phytoremediation. Also, *P. perfoliatus* proved to be a reliable indicator for the presence of PTEs. In addition, the mosses *C. fontinaloides* and *F. antipyretica* can be used as stenovalent species as indicators of good ecological status in large rivers with predominantly stony/gravelly substrates.

The data obtained by the analysis of relations between PTE contaminants and the diversity and distribution of macrophytes communities increases our understanding of PTE contamination effects on aquatic plant communities in the Sava River Basin.

## Data availability statement

The data obtained in this study will be available on request.

## Author declaration

### Submission declaration

This article is original and has not been previously published, in whole or in part. This work is not under consideration by any other journal elsewhere and its publication is approved by all authors. In case of the acceptance of this paper, it will not be published elsewhere in the same form, in English or any other language, including electronically without the written consent of the copyright holder.

### Authorship statement

We confirm that the manuscript has been read and approved by all named authors. We confirm that the order of authors listed in the manuscript has been approved by all named authors.

## CRediT authorship contribution statement

**Snežana Jarić:** Writing – review & editing, Writing – original draft, Conceptualization. **Branko Karadžić:** Writing – review & editing, Writing – original draft. **Momir Paunović:** Methodology, Investigation, Formal analysis. **Radmila Milačič:** Methodology, Investigation, Formal analysis. **Janez Ščančar:** Methodology, Investigation, Formal analysis. **Olga Kostić:** Visualization, Investigation. **Tea Zuliani:** Methodology, Investigation, Formal analysis. **Janja Vidmar:** Methodology, Investigation, Formal analysis. **Zorana Miletić:** Writing – review & editing, Validation, Formal analysis. **Stefan Anđus:** Methodology, Investigation, Formal analysis. **Miroslava Mitrović:** Writing – review & editing, Validation, Formal analysis. **Pavle Pavlović:** Resources, Funding acquisition.

## Declaration of competing interest

The authors declare that they have no known competing financial interests or personal relationships that could have appeared to influence the work reported in this paper.
